# Pars plana vitrectomy and subretinal tissue plasminogen activator for large exudative submacular hemorrhage: a case series

**DOI:** 10.1186/s12886-022-02639-w

**Published:** 2022-10-27

**Authors:** Direk Patikulsila, Pawara Winaikosol, Janejit Choovuthayakorn, Nawat Watanachai, Voraporn Chaikitmongkol, Paradee Kunavisarut

**Affiliations:** Department of Ophthalmology, Faculty of Medicine, Chiang Mai University, 110 Intavaroros Road, Maung, Chiang Mai, 50200 Thailand

**Keywords:** Large submacular hemorrhage, Pars plana vitrectomy, Tissue plasminogen activator, Exudative macular degeneration

## Abstract

**Background:**

To evaluate anatomical and functional outcomes of patients with large submacular hemorrhage (SMH) who treated by pars plana vitrectomy (PPV) in combination with subretinal tissue plasminogen activator (TPA) injection, intraocular gas tamponade, and with additional post-operative interventions.

**Methods:**

Medical records of 9 patients who presented with large SMH secondary to age-related macular degeneration (AMD) and underwent PPV, subretinal TPA injection, and gas tamponade at Chiang Mai university hospital between January 2012 and January 2020 were reviewed. Collected data included preoperative visual acuity (VA), SMH extent and duration, intraoperation and post-operation complications, post-operative anatomical and VA responses, and the need for administer post-operation additional treatments.

**Results:**

Overall, five patients were male and four patients were female with a mean (SD) age of 66.9 (7.7) years and a mean (SD) follow-up of 21.1 (16.1) months. A mean (SD) duration of SMH was 15.1 (10.9) days with a mean (SD) extent of SMH was 6.2 (3.4) disc diameters. At 1-month post-operation, complete SMH displacement was noted in eight (88.9%) patients. The mean (SD) VA significantly improved from LogMAR 1.9 (0.4) to 1.1 (0.4), (*P* = 0.004). During follow-up, eight patients (88.9%) were given additional therapy (anti-vascular endothelial growth factor (anti-VEGF) monotherapy, photodynamic therapy, or in combination). At final follow-up, a mean (SD) LogMAR VA of 0.9 (0.4) was significantly improved compared to baseline (*P* = 0.004). For intra- and post-operation complications, none developed intraoperative retinal break and retinal detachment.

**Conclusions:**

Vitrectomy with subretinal TPA injection, intraocular gas tamponade, and additional post-operation treatments provide benefit for anatomical and visual outcomes for patients with large SMH. It may consider as one of effective treatment in this group of patients.

## Background

Based on evidence from many randomized controlled trials, the current mainstay treatment for neovascular age-related macular degeneration (AMD) is intravitreal anti-vascular endothelial growth factor (anti-VEGF) injection [1–7]. However, for an uncommon neovascular AMD complicated with a large submacular hemorrhage (SMH), the optimal therapeutic approaches remain uncertain. In this subgroup, an accumulation of blood underneath a foveal center could increase the risk of irreversible photoreceptor and outer retinal damage. Previous studies reported that if left untreated, a high proportion of patients with large SMH developed permanent and severe visual impairment [8, 9].

Due to a lack of convincing evidence from comparative studies, various treatment strategies were investigated as either mono- or combination therapy. These maneuvers included pneumatic displacement, intravitreal or subretinal tissue plasminogen activator (TPA) injection, using tamponade agents (including perfluorocarbon liquid (PFCL), silicone oil, or gas), subretinal surgery for blood clot removal, intravitreal or subretinal anti-VEGF injection, and photodynamic therapy (PDT) [10–22]. Therefore, the visual and anatomical responses were variably reported between publications.

This study aims to describe outcomes of patients with large SMH secondary to exudative AMD who underwent pars plana vitrectomy (PPV), subretinal TPA injection, or/and gas tamponade, with/or without postoperative additional interventions in a single tertiary referral center.

## Methods

This retrospective observational case series was approved and the informed consent was waived by the Research Ethics Committee, Faculty of Medicine, Chiang Mai University, Thailand. The protocol was conducted in accordance with the Declaration of Helsinki. We identified medical and operative records of consecutive patients diagnosed with large SMH, at least 4-disc diameter, secondary to exudative AMD, who underwent PPV, subretinal TPA injection, and gas tamponade at Chiang Mai University Hospital between January 2012 and January 2020. All patients with at least one post-operative follow-up were included.

### Surgical techniques and post-operative posturing

The operations were performed under local anesthesia using a 3-port 23-gauge vitrectomy system. The procedures were initiated by core vitrectomy, posterior hyaloid induction, and peripheral shaving. Then, a 39-gauge subretinal injection cannula tip (Synergetics, USA) was placed into the subretinal space, and TPA (concentration of 100 µg in 1 ml) [23] was injected by a viscous fluid controlling system. The volume and amount of TPA administered varied according to the extent and thickness of the SMH but was within a range of 25 to 50 µg (to detach the retina from the hemorrhage and cover the entire hemorrhage area). After that, air-fluid and gas-air exchange was partially performed. At the end of surgery, intravitreal anti-VEGF injection was administered at the physician’s discretion. The operations were performed by an experienced vitreoretinal surgeon (DP). After the surgery, the patients were instructed to lie down on their backs for 30 min to allow clot liquefaction [21, 24]. Then, the patients were asked to perform chin down 45 degrees positioning for 12 h, followed by face down positioning until the gas was gone. Additional postoperative treatments were given *pro re nata* at the discretion of the treating physician.

The data were reviewed for age, gender, systemic comorbidities, and duration of visual loss. In addition, at baseline and each follow-up visit, ophthalmic data including Snellen visual acuity (VA), anterior and posterior segment findings by slit-lamp biomicroscopy, color fundus photography, fluorescein (FFA) and indocyanine green angiography (ICGA), and optical coherence tomography (OCT), if available, were collected.

### Statistical analysis

Descriptive statistics were used to analyze the data and reported as mean (SD) and range for continuous data and percentage for categorical data. Snellen VA was converted to the logarithm of the minimum angle of resolution (LogMAR) and assigned light perception as 2.7 LogMAR, hand movement as 2.3 LogMAR, and counting finger as 1.9 LogMAR [25].

At month 1 post-operation, displacement of SMH was categorized based on color fundus photograph and fundus examination into complete displacement (scarce amount of blood within one disc diameter of foveal center), partial displacement (visible amount of blood underneath the fovea with retinal elevation), and no displacement (unchanged blood amount underneath the fovea) [26]. Pre- and post-operative VA comparison was performed by the Wilcoxon signed-rank test. Statistical significance was set at P value less than 0.05. Data analysis was performed using STATA version 16.0 software.

## Results

Overall, there were nine patients (5 male and 4 female) with a mean (SD) age of 66.9 (7.7) years and a mean (SD) follow-up of 21.1 (16.1) months (range, 3 to 38 months) included in this study. All patients had large SMH involving the fovea and were treatment naïve. None were taking systemic anticoagulant medications. The mean (SD) presenting LogMAR VA was 1.9 (0.4) (Snellen equivalent 20/1588). The mean (SD) duration of symptoms before surgery was 15.1 (10.9) days (range, 4 to 30 days) with four patients (44.4%) presenting later than 14 days. The mean (SD) extent of SMH was 6.2 (3.4) disc diameter (DD) (range, 4 to 15 DD). Two patients (22.2%) were noted as pseudophakia.

Intraoperatively, non-expansile sulfurhexafluoride (SF6) was used as a tamponade in seven (77.8%) patients, while non-expansile perfluoropropane (C3F8) was used in two (22.2%) patients. At the end of surgery, intravitreal bevacizumab was administered to five (55.6%) patients.

At one-month post-operation, based on additional information from cross-sectional OCT, FFA, and/or ICGA, five patients (55.6%) were diagnosed with polypoidal choroidal vasculopathy (PCV) when a branching neovascular network (BNN) lying between retinal pigment epithelium (RPE) and Bruch’s membrane, a sharp-peaked pigment epithelium detachment (PED), and a hyperfluorescence spot corresponding to polypoidal lesion were detected. Among the remaining patients, three (33.3%) were diagnosed with AMD when BNN without an accompanying complex or multilobular PED were observed. Of note, data for one patient could not be obtained. Vision significantly improved to a mean (SD) LogMAR VA of 1.1 (0.4) (Snellen equivalent, 20/251) compared to before surgery (*P* = 0.004). Displacement of SMH was categorized as complete in eight (88.9%) patients (three out of four patients who presented > 14 days and all of five patients who presented within 14 days from symptoms). One (11.1%) patient had partial SMH displacement. During follow-up with angiography and OCT, eight patients (88.9%) were given additional therapy: six received intravitreal anti-VEGF injections, one received combination PDT with intravitreal anti-VEGF injections, and one received PDT monotherapy. At final follow-up, a mean (SD) LogMAR VA of 0.9 (0.4) (Snellen equivalent, 20/158) was significantly improved compared to before surgery (*P* = 0.004), but there was a non-significant difference compared to post-operative month 1 (*P* = 0.063). Seven patients (77.8%) achieved a VA of 20/200 and better. Table 1 summarizes baseline characteristics of each patient.

**Table 1:** Characteristics of patients with large submacular hemorrhage secondary to exudative age-related macular degeneration

Case	Age (Y)	Sex	Eye	SMH		Dx	Operation		Displacement of SMH at Month 1	Post- Op	Snellen Visual Acuity		
				**Duration (Day)**	**Extent (DD)**		**Gas**	**Anti-VEGF**			**Baseline**	**Month 1**	**Final**
1	62	M	OD	7	5.5	PCV	C3F8	BZB	Complete	Anti-VEGF	20/400	20/200	20/80
2	68	M	OS	4	4.2	PCV	SF6	BZB	Complete	Anti-VEGF, PDT	CF	20/200	20/60
3	78	F	OD	4	5.6	AMD	C3F8	No	Complete	No	HM	20/200	20/1200
4	63	M	OS	7	4.0	N/A	SF6	No	Complete	Anti-VEGF	HM	20/200	20/200
5	76	F	OS	30	6.6	AMD	SF6	No	Partial	Anti-VEGF	CF	20/200	20/200
6	72	F	OD	30	5.2	PCV	SF6	No	Complete	PDT	20/400	20/200	20/200
7	54	M	OS	21	15.0	PCV	SF6	BZB	Complete	Anti-VEGF	HM	20/200	20/60
8	68	F	OD	9	5.0	PCV	SF6	BZB	Complete	Anti-VEGF	CF	20/120	20/60
9	61	M	OD	23	5.1	AMD	SF6	BZB	Complete	Anti-VEGF	HM	20/400	20/400

*Y* year, *SMH* submacular hemorrhage, *DD* disc diameter, *Dx* diagnosis, *Anti-VEGF* anti-vascular endothelial growth factor, *M* male, *F* female, *OD* right eye, *OS* left eye, *PCV* polypoidal choroidal vasculopathy, *AMD* age-related macular degeneration, *C3F8* perfluoropropane, *SF6* sulfurhexafluoride, *BZB* bevacizumab, *CF* counting finger, *HM* hand movement, *PDT* photodynamic therapy

**Fig. 1:**
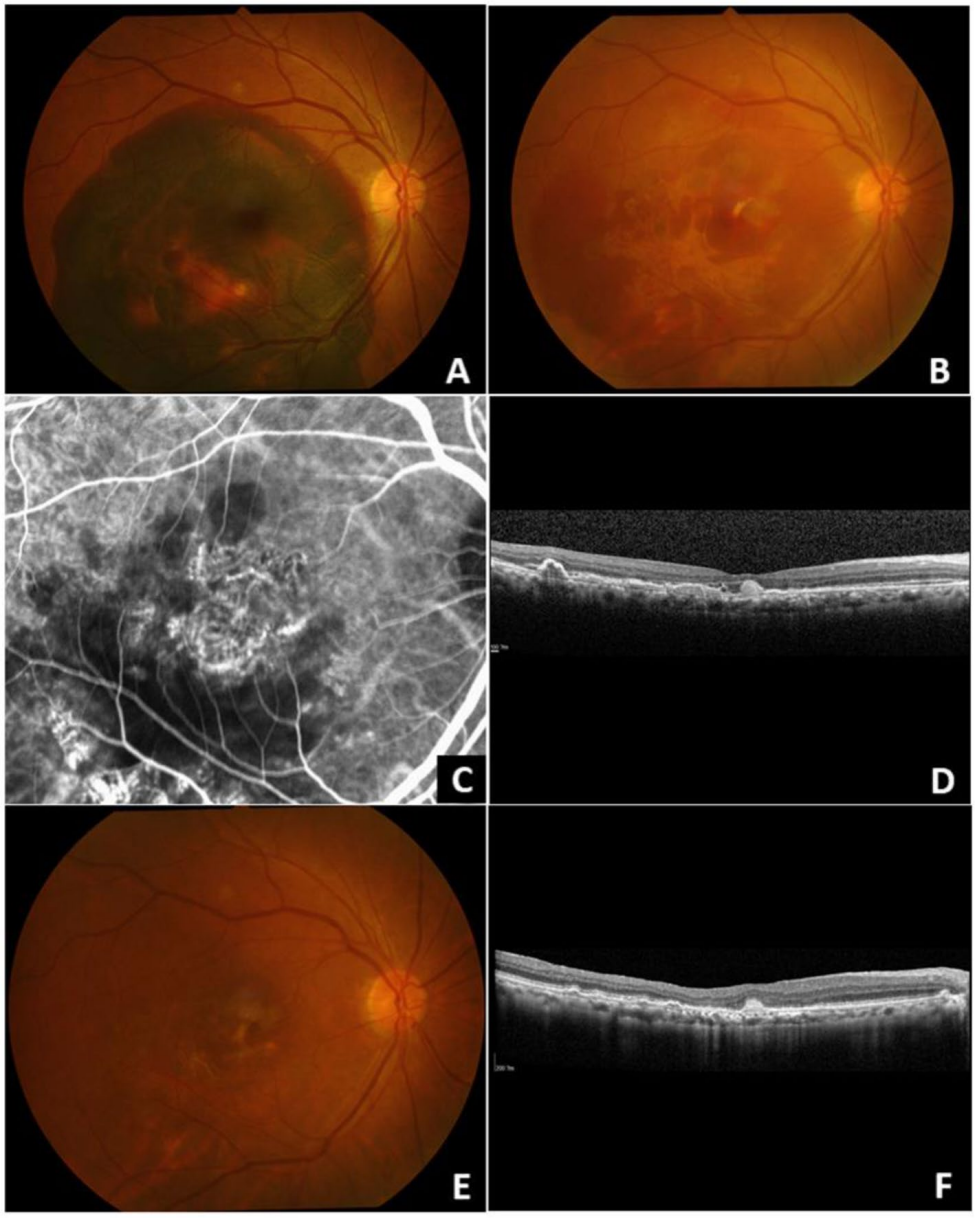
A 62-year-old Thai male presented with decreased vision in his right eye for 7 days (case 1). Color fundus photograph at initial presentation shows a large submacular hemorrhage (SMH) measuring 5.5-disc diameter in size (**A**). One month after pars plana vitrectomy with subretinal tissue plasminogen activator injection and fluid-gas exchange, a partial displacement of SMH is noted (**B**). Indocyanine green angiography reveals a branching vascular network underneath a foveal area (**C**). Optical coherence tomography (OCT) reveals branching neovascular network lying between retinal pigment epithelium and Bruch’s membrane and multilobular sharp-peaked retinal pigment epithelial detachment (**D**). At the final visit, submacular fibrosis is shown on the color fundus photograph (**E**) and regression of sharp-peaked PED is observed on the OCT following intravitreal anti-vascular endothelial growth factors injections (**F**)

**Fig. 2:**
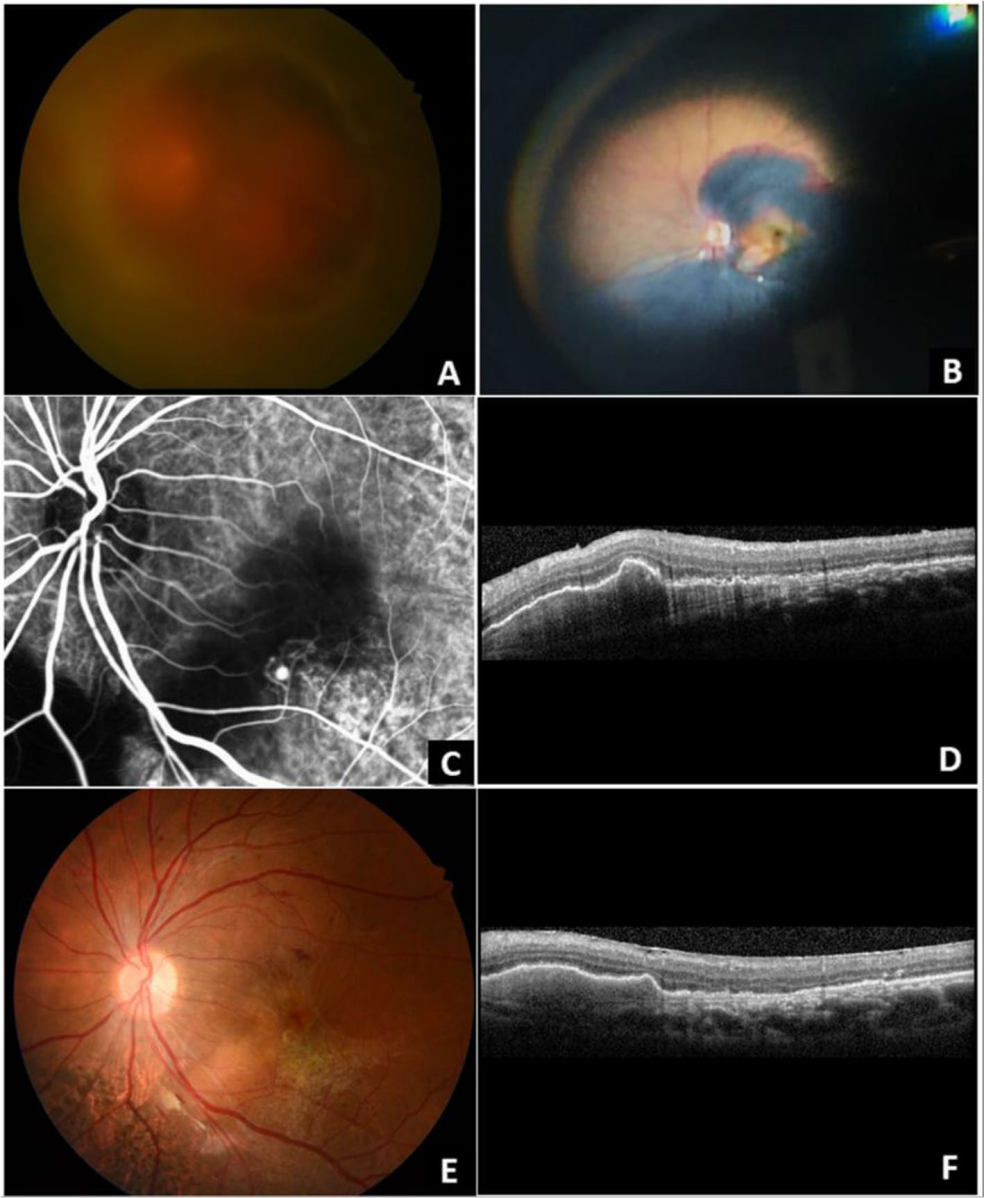
A 54-year-old Thai male presented with blurred-vision in his left eye for 21 days. A breakthrough vitreous hemorrhage accompanied by submacular hemorrhage (SMH) is observed on fundus examination (**A**). Intraoperatively, a large thick SMH is found (**B**). At one month after pars plana vitrectomy, a hyperfluorescence spot corresponding to polypoidal lesion is observed on indocyanine green angiography (**C**). Optical coherence tomography (OCT) reveals a sharp-peaked retinal pigment epithelial detachment with subretinal pigment epithelial ring-like lesions (**D**). At the final follow-up, macular fibrosis is noted over the previous hemorrhage area on color the fundus photograph (**E**) and partial regression of a sharp-peaked PED is noted on the OCT following intravitreal anti-vascular endothelial growth factors injections (F)

Regarding intraoperative complications, none developed retinal and retinal pigment epithelium (RPE) tear. One patient (case 1) developed an early post-operative hemorrhage in the anterior chamber and vitreous cavity requiring a PPV and washing procedure. During follow-up, three of seven phakic patients had progressions of lens opacities and underwent cataract surgery. One patient (case 8) developed a recurrence of a large SMH and PPV with subretinal TPA injection was re-performed at month 36 after the initial surgery. One patient (case 1) developed epiretinal membrane. Other complications, including rhegmatogenous retinal detachment, macular hole, and high intraocular pressure were not observed. Figure 1 and 2 illustrate the baseline and clinical results of 2 patients with large SMH who underwent PPV and subretinal TPA injection in this study.

## Discussion

Large SMH is one of the uncommon manifestations of several retinal and choroidal diseases but mainly accompanies AMD. Various therapeutic approaches have been described to manage this condition. This study reviews the efficacy of PPV, subretinal TPA injection, and pneumatic displacement, with or without additional postoperative treatments to successfully remove a subfoveal hemorrhage and sustain visual improvement in patients with a large SMH secondary to exudative AMD.

At present, even with advances in medical innovation, the management of SMH complicating exudative AMD remains challenging. In this condition, an accumulation of subretinal blood may further accelerate RPE and photoreceptor damage beyond a preexisting destruction caused by a neovascular complex. Therefore, to minimize the risk of permanent and severe visual and structural alterations, several treatment strategies have been reported to clear and/or displace blood clots away from the foveal center and to minimize disease activity of underlying etiologies [18–20, 27, 28].

Surgical-based intervention by vitrectomy is one of the treatment options that has been widely investigated. However, with differences in patients and lesion characteristics, surgical techniques and adjunctive procedures such as post-operative intravitreal anti-VEGF injection, various outcomes were noted. In the Subretinal Surgery Trial, patients with predominantly hemorrhagic subfoveal choroidal neovascularization who underwent PPV to mechanically remove subretinal blood clots and/or neovascular complexes showed no significant difference in stabilization or vision improvement compared to observation [9].

Nevertheless, for patients with a massive bullous subretinal hemorrhage (SRH) exceeding two or more quadrants of peripheral retina, either with or without complicating vitreous hemorrhage, the efficacy of PPV for improving vision has been demonstrated in several studies [22, 29, 30]. Among those, Oshima et al. reported that by undergoing pre-operative intravitreal TPA injection with subsequent PPV, small peripheral retinotomy, PFCL, SMH drainage, and long-acting gas tamponade, 88% of patients had visual improvement, and all of these improving eyes achieved a final VA of 20/400 or better [22]. Likewise, Wei et al. [29] reported that following PPV with 360-degree retinotomy, PFCL displacement, and silicone oil tamponade, 90% of patients with massive SMH achieved visual improvement, and 52% of patients attained a visual acuity of 20/400 or better. Additionally, anatomical success following two-stage PPV with subretinal TPA and PFCL tamponade for displacing massive SMH involving subretinal and sub-RPE hemorrhage has also been described [31].

For large SMH, PPV combined with subretinal TPA injection and intraocular gas tamponade was one of the frequent procedures performed with a relatively high success rate, ranging from 64 to 100%, for complete subretinal hemorrhage displacement [20, 21, 24, 32–34]. Several influencing factors for the efficacy of PPV and subretinal TPA for large SMH were investigated including the extent and thickness of SMH, duration of symptoms, and baseline VA; however, the controversial associations remain [24, 32, 35–38]. Jeong et al. [33] explored associations between treatment modalities, lesion size, and responses for patients with SMH. Their study compared three treatment options (intravitreal anti-VEGF injection, pneumatic displacement combined with anti-VEGF injection, and PPV with subretinal TPA and gas tamponade), and found favorable outcomes for patients having small lesions (less than four-disc diameters) regardless of treatment groups. However, in patients with large SMH, better results were shown in patients who underwent PPV compared to patients in the pneumatic displacement group.

Our study confirmed a promising efficacy of PPV combined with TPA and gas tamponade for displacing subretinal blood in patients presenting with a large SMH. Complete displacement was achieved in almost 90% of patients within one month. This may partly refer to a short presentation period, a maximum of 30 days, for patients in this study. However, the use of intraocular gas tamponade with a long duration of face down positioning as described in this study might be one of the significant barriers for patients undergoing PPV. A 40-degree gaze down positioning over 1 to 2 weeks post-operation might facilitate better blood displacement and be better tolerated by patients [39]. In addition to anatomical success, few operative complications were encountered in this study.

Regarding visual outcome, the role of intra and/or post-operative intravitreal anti-VEGF injection, theoretically, offers potential benefit for treating underlying neovascular complex without being delayed by an obscuring detailed fundus view. Chang et al. [26] demonstrated that patients with large SMH who underwent PPV without receiving post-surgical anti-VEGF treatment had vision improvement in early postoperative visits, but the amount of visual gain slightly declined over time. However, maintenance of improved vision in patients who received post-operative anti-VEGF treatment was observed throughout the study period.

A progression of underlying conditions and/or a recurrence of SMH may relate to several factors. The ability to control pathological processes might play a role in maintaining post-operative vision. In our study, based on the discretion of treating physicians, further post-operative treatments with anti-VEGF and/or PDT were given to eight patients. In this group, the maintenance of improved vision was observed during the follow-up period. Recurrence of SMH occurred in 1 patient; however, it was successfully managed by re-operation. One patient who had no active intra/subretinal fluid and did not receive post-operative anti-VEGF treatment during follow-up visits, gradually developed submacular scarring and decreased visual gain at the final visit. Nevertheless, with the small sample size, the impact of associated factors related to disease progression could not be explored.

Limitations of this study included the retrospective nature of this study that contained variations in follow-up duration and post-operative treatments. In addition, though encouraging results were noted, the efficacy of PPV could not compare to other surgical techniques and/or other treatment modalities. However, this study supports the evidence that in patients with large SMH complicating exudative AMD, the high proportion of vision improvement with a relatively low incidence of serious postoperative complications could be achieved following PPV, subretinal TPA injection, and gas tamponade, with additional PDT and /or anti-VEGF therapy. The results from several ongoing comparative clinical trials are warranted.

## Conclusions

PPV, subretinal TPA injection, intraocular gas tamponade, and additional post-operative treatments seem valuable for patients with large SMH in terms of an early subfoveal hemorrhage displacement and sustaining post-operative visual gain.

## Data Availability

The datasets analyzed in the current study are available from the corresponding author on reasonable request.

## Abbreviations

AMD: Age-related macular degeneration
Anti-VEGF: Anti-vascular endothelial growth factor
C3F8: Perfluoropropane
FFA: Fluorescein angiography
ICGA: Indocyanine green angiography
LogMAR: Logarithm of the minimum angle of resolution
OCT: Optical coherence tomography
PCV: Polypoidal choroidal vasculopathy
PDT: Photodynamic therapy
PFCL: Perfluorocarbon liquid
PPV: Pars plana vitrectomy
RPE: Retinal pigment epithelium
SD: Standard deviation
SF6: Sulfurhexafluoride
SMH: Submacular hemorrhage
TPA: Tissue plasminogen activator
VA: Visual acuity
